# Field triage in trauma – do the data really justify the conclusions?

**DOI:** 10.1186/1757-7241-17-24

**Published:** 2009-05-29

**Authors:** Mårten Sandberg

**Affiliations:** 1 Air Ambulance Department, Oslo University Hospital – Ullevål, Oslo, Norway

## Letter

Dear Sir,

I read with interest the recently published paper by Rehn and coworkers about field triage in trauma [1]. The topic is interesting and improved quality of the work and information flow from the scene-of-the-accident to the emergency department can save lives. However, some of the conclusions drawn by the authors can be challenged.

First, the authors compared undertriage and overtriage of the traumatized patients and found 2% and 17% undertriage and 35% and 66% overtriage for anaesthetists and paramedics, respectively. They conclude that "anaesthetists perform precise trauma triage, whereas paramedics have potential for improvement" although the authors themselves state that "skewed mission profiles make comparison of differences in triage precision difficult" [1]. The ground ambulances staffed with paramedics are used locally while the helicopters staffed with anaesthetists are a regional resource. The helicopters are dispatched when major trauma is suspected while ground ambulances are dispatched to any sort of incidence. In Oslo, an anaesthetist-staffed ground ambulance operates alongside ordinary ambulances and the patients transported with this service are a subgroup of the patients transported by anaesthetists. If the triage precision between paramedics and anaesthetists is to be compared, data from ground ambulances in Oslo (with or without anaesthetist) should be used and the data from patients brought to the hospital by helicopter or other services should be excluded. Such a comparison would give a good indication about the real difference in triage precision between the two groups of prehospital care providers. Unfortunately, that subgroup analysis has not been performed. That is sad, because the numbers that is provided in the article is of little interest since the services that are compared are too different.

Second, in the system described, the paramedics or the anaesthetists examine the patient and investigate the mechanism of the accident before reporting the findings and the patient's symptoms either directly to the hospital or to the dispatch centre. The emergency room nurse who receives the pre-notification call decides whether or not to activate the trauma team based upon given predefined criteria. That way, field triage as such concerning trauma team activation, does not exist in this system. The ED nurse activates the trauma team. The title of the paper is thus misleading.

Third, is the reported overtriage or undertriage the result of erroneous information from the field, incorrect interpretation of the prehospital information by the dispatch centre or the ED nurse or does it result from trauma team activation guidelines that are not precise? In the present study, the published data is not detailed enough to determine what is actually the reason(s) for the over- and undertriaged patients. A correct field report can, depending on the circumstances, result in both undertriage, overtriage or correct triage.

Further, in the paper [1], it is stated that the anaesthetists' undertriage is 2%. It is a low figure and as such sounds acceptable, but what does it really tell? In the system described, the trauma team is activated in the huge majority of cases when the helicopter anaesthetist is bringing patients to the hospital independent of whether the anaesthetist has specifically requested TTA or not. It is not reported which fraction of the patients brought to UUH by an anaesthetist were brought there by helicopter, but it is probably the majority. It is very difficult to get a significant undertriage when the trauma team is activated in almost all the cases. Furthermore, what is the correct definition of undertriage when it comes to the helicopter anaesthetists? A severely injured patient who is brought to a local hospital since the helicopter anaesthetist misinterpreted the patient's condition will not be included in the study. This will lead to an artifically low undertriage for the anaesthetists. The real undertriage of helicopter anaesthetist patients is not known.

Finally, and the most disturbing information in Rehn's report [1] is that the patients subjected to undertriage had a higher 30-day mortality (adjusted odds ratio 2.34) than patients that were initially correctly triaged. It seems reasonable to assume that it takes some time (hours?) from patient arrival at the hospital till the severity of the patient's condition is recognized for an increased mortality to result. Clearly, the in-hospital patient treatment cannot and should not be dictated by the prehospital prenotification about the patient's condition and the trauma mechanism. Assuming that the numbers reported in Rehn's paper as well as the statistical handling of them are correct, the report indicates that UUH has a potential for improved identification of severly injured patients when the trauma team is not initially activated.

Consequently, I challenge the authors' conclusion that "anaesthetists perform precise trauma triage, whereas paramedics have potential for improvement". I believe the data do not justify the conclusion. Their data shows that the whole ***chain ***consisting of prehospital examination and hospital prenotification, trauma team activation and trauma team activation guidelines should be improved. However, the study presented here is not designed to identify which of the links of the chain that are weak. Hopefully, this will be addressed in future studies.

## References

[B1] Rehn M, Eken T, Krüger AJ, Steen PA, Skaga NO, Lossius HM (2009). Precision of field triage in patients brought to a trauma centre after introducing trauma team activation guidelines. Scand J Trauma Resusc Emerg Med.

